# Case Report: Early Resection of Pheochromocytoma in a Patient With Cardiogenic Shock Due to Pheochromocytoma-Induced Cardiomyopathy With Extracorporeal Life Support

**DOI:** 10.3389/fcvm.2022.788644

**Published:** 2022-03-21

**Authors:** Ting Lyu, Jianhua Niu, Zhihai Liu, Tong Li

**Affiliations:** Department of Intensive Care Medicine, The First Affiliated Hospital, College of Medicine, Zhejiang University, Hangzhou, China

**Keywords:** pheochromocytoma-induced cardiomyopathy, cardiogenic shock, veno-arterial extracorporeal membrane oxygenation, case report, cardiac arrest

## Abstract

**Background:**

Pheochromocytoma-induced cardiomyopathy is a rare but potentially life-threatening complication of pheochromocytoma. It mimics the patterns of stress-induced cardiomyopathy. In severe cases, patients can develop refractory cardiogenic shock, which might require mechanical circulatory support.

**Case Presentation:**

We presented a case of 54-year-old female who developed refractory cardiogenic shock, following an elective orthopaedic surgery complicated by cardiac arrest, requiring veno-arterial extracorporeal membrane oxygenation (VA-ECMO) support. After urgent coronary catheterisation revealed normal coronary arteries, further evaluation of the aetiology of cardiogenic shock revealed pheochromocytoma. With a diagnosis of pheochromocytoma-induced cardiomyopathy, the patient had accelerated preoperative alpha adrenergic blockade preparation for a total of 6 days and subsequently had the tumour removed under VA-ECMO support. Postoperatively, the patient recovered well and was off ECMO support and extubated a few days later.

The optimal management of pheochromocytoma-induced cardiomyopathy, especially for severe cases, is still unclear. Indeed, some cases will require mechanical circulatory support to allow left ventricular function recovery. But our case also showed that it was possible to introduce alpha blockade safely whilst the patient is on VA-ECMO and has the pheochromocytoma removed with VA-ECMO support after accelerated preoperative preparation.

## Background

Pheochromocytomas are rare but potentially life-threatening tumours, arising from chromaffin cells of the adrenal medulla or extra-adrenal paraganglia. Most patients present with common symptoms, such as episodic headache, sweating, and tachycardia, whilst, occasionally, some patients present with a pheochromocytoma crisis. Uncontrolled catecholamine release can lead to catastrophic consequences, such as myocardial dysfunction. With a pattern similar to stress-induced cardiomyopathy, which is characterised by transient regional systolic dysfunction of the left ventricle (commonly apical and midventricular aknesis or dyskinesis and hyperkinesis of the base) without angiographic evidence of obstructive coronary artery disease or acute plaque rupture, pheochromocytoma-induced cardiomyopathy can be misdiagnosed as stress-induced cardiomyopathy (1, 2). Here, we present a case of a middle-age female with pheochromocytoma-developed refractory cardiogenic shock required veno-arterial extracorporeal membrane oxygenation (VA-ECMO) support, following an elective orthopaedic surgery. Subsequently, the patient successfully underwent urgent pheochromacytoma resection under VA-ECMO support.

## Case Presentation

**Figure 1:**
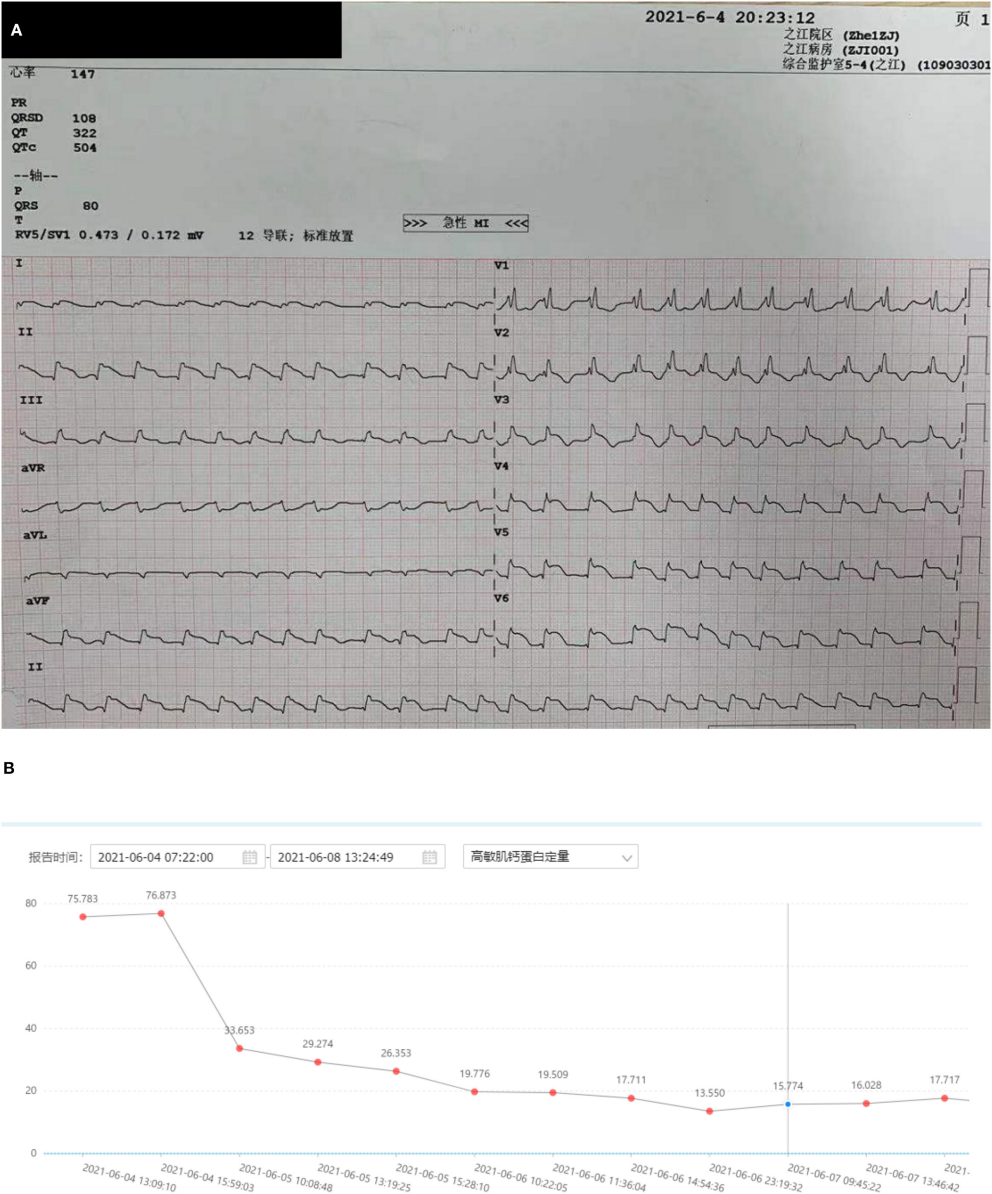
Electrocardiogram of the patient. **(A)** Electrocardiogram of the patient (arrow: ST segment elevation). **(B)** A trend of troponin I (nanograms per litre).

A 54-year-old Chinese female without significant past medical history underwent an elective right shoulder arthroscope under general anaesthesia on the 3rd of June 2021 in a rural hospital. During her outpatient orthopaedic follow-up, her blood pressure was 128/68 mmHg with a heart rate of 78 beats per minute. Intra-operatively and immediate post-operatively, she had stable hemodynamic status. However, she developed symptoms including palpitation, chest pain, and sweating the same night; the vital signs check revealed asystolic blood pressure of 170 mmHg with a heart rate of 100 beats per minute. On physical examination, the patient was still alert and conscious, but diaphoretic and tachypneic with a respiratory rate of 35 per minute. There were bilateral crepitations on auscultation. Her heart sounds were normal; extremities were cool. Abdominal examination was unremarkable. There was no pedal oedema. The electrocardiogram (ECG) revealed right bundle branch block and widespread ST-segment elevation at inferior and lateral leads (Figure 1). Urgent cardiology consultation was made. The impression from cardiologist was ST-segment elevated myocardial infarction (STEMI). Whilst preparing for urgent coronary angiogram, the patient rapidly deteriorated. She became cyanotic and hypotensive. She was intubated and supported with a high dose of inotropes. Bedside echocardiogram showed depressed left ventricular systolic function with left ventricular ejection fraction (LVEF) of 20%, but preserved right ventricular function with tricuspid annular plane systolic excursion (TAPSE) of 18 mm. Despite all support, the patient developed ventricular fibrillation and had return of spontaneous circulation (ROSC) after 3 min of resuscitation. The patient was reassessed by a cardiologist in the local hospital after the rapid deterioration and was deemed too unstable for coronary intervention without mechanical circulatory support. Hence, she was referred to our hospital for consideration of urgent revival with VA-ECMO for refractory cardiogenic shock. After tele-consultation by the ECMO specialist in our hospital, the patient was accepted for VA-ECMO support. The patient was supported by 1.5 μg/kg/min of noradrenaline and 0.5 μg/kg/min of adrenaline by the time our ECMO retrieval team arrived. She was then put on VA-ECMO support with adrenaline weaned off and noradrenaline decreased down to 0.3 μg/kg/min and subsequently transferred to our hospital for further management.

**Figure 2:**
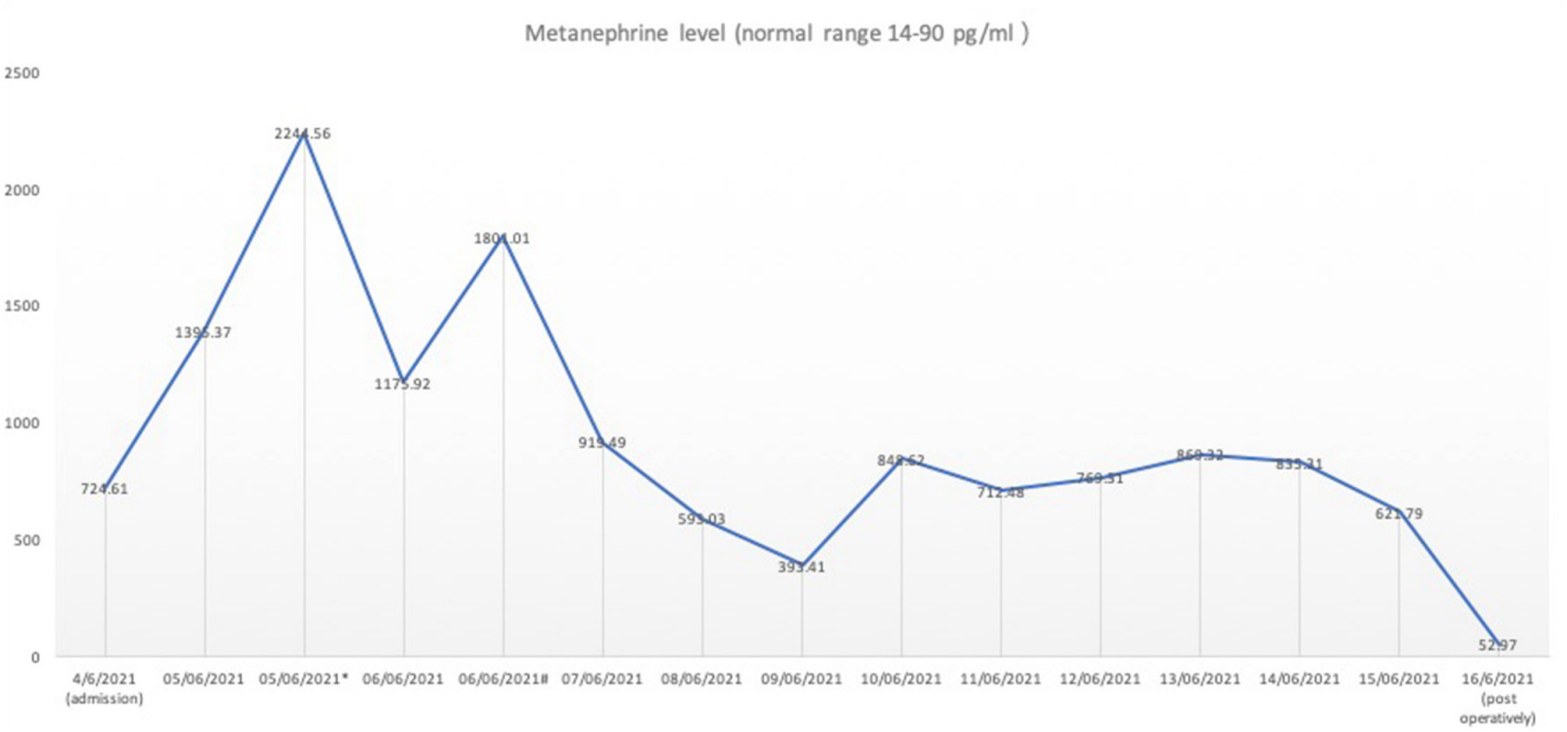
The serum metanephrine level pre-operatively and post-operatively. The serum metanephrine level was measured continuously at 6 a.m. from 5th June 2021 onwards. *The metanephrine level during the first episode of the sudden onset of hypertension and tarchycardia. ^#^The metanephrine level during the second episode of the sudden onset of hypertension and tarchycardia. On 16th June 2021, the metanephrine level dropped back to the reference range post-operatively on Day 1.

**Figure 3:**
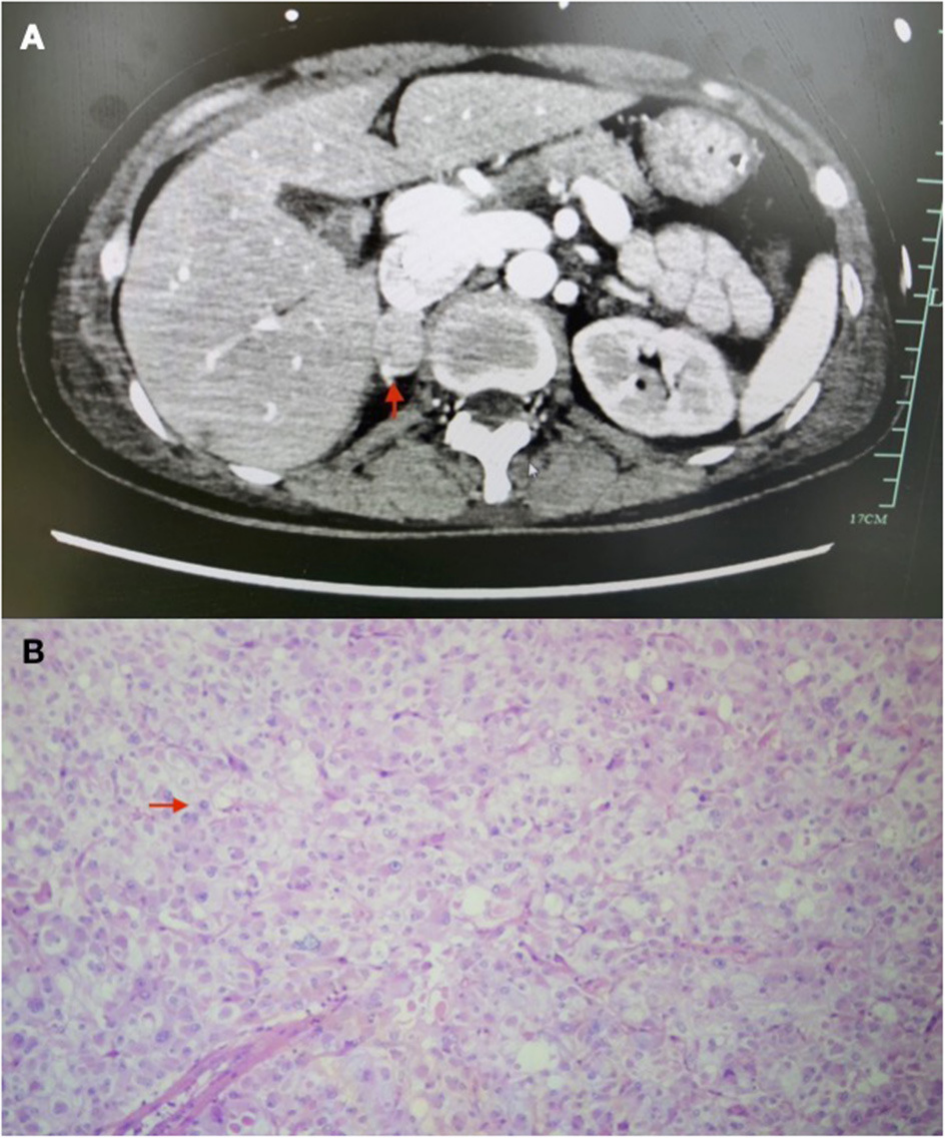
Images of adrenal tumour. **(A)** CT images of adrenal tumour (arrow). **(B)** Histology image of adrenal tumour (arrow: characteristic-stippled chromatin).

The patient underwent an urgent coronary angiography on arrival to our hospital, which, surprisingly, was normal, notwithstanding the typical ECG changes and raised troponins. Given the severe myocardial dysfunction with a normal coronary angiogram, we had a detailed review of her past medical history with the patient's daughter. The patient's daughter denied any significant medical condition including any cardiac issues and hypertension and confirmed that the patient had normal effort tolerance pre-operatively. But she mentioned that the patient had a cardiac workup done in June of last year due to palpitations and breathlessness, which included a normal echocardiogram and normal coronary angiogram. Also, the patient forgot to follow up her adrenal nodule, which was picked up incidentally during a previous health screening. Based on the new information, together with the patient's acute history and past investigations, suspicion of pheochromocytoma-induced cardiomyopathy was raised. The vasopressor strategy was changed to non-catecholamine-based drugs, including vasopressin and levosemendan. As the patient was anuric and still on noradrenaline infusion when pheochromocytoma-induced cardiomyopathy was suspected, serial serum samples for the metanephrine level were sent (Figure 2). Contrast adrenal CT was done, showing a 21 mm × 16 mm × 10 mm heterogeneous mass with regular spherical, smooth margins, and enhancement during the arterial phase (Figure 3A). Also, the patient had episodes of a sudden onset of hypertension and tachycardia without any stimulus and spontaneously resolved minutes later. The plasma metanephrine level was measured during these episodes also (Figure 2).

**Figure 4:**
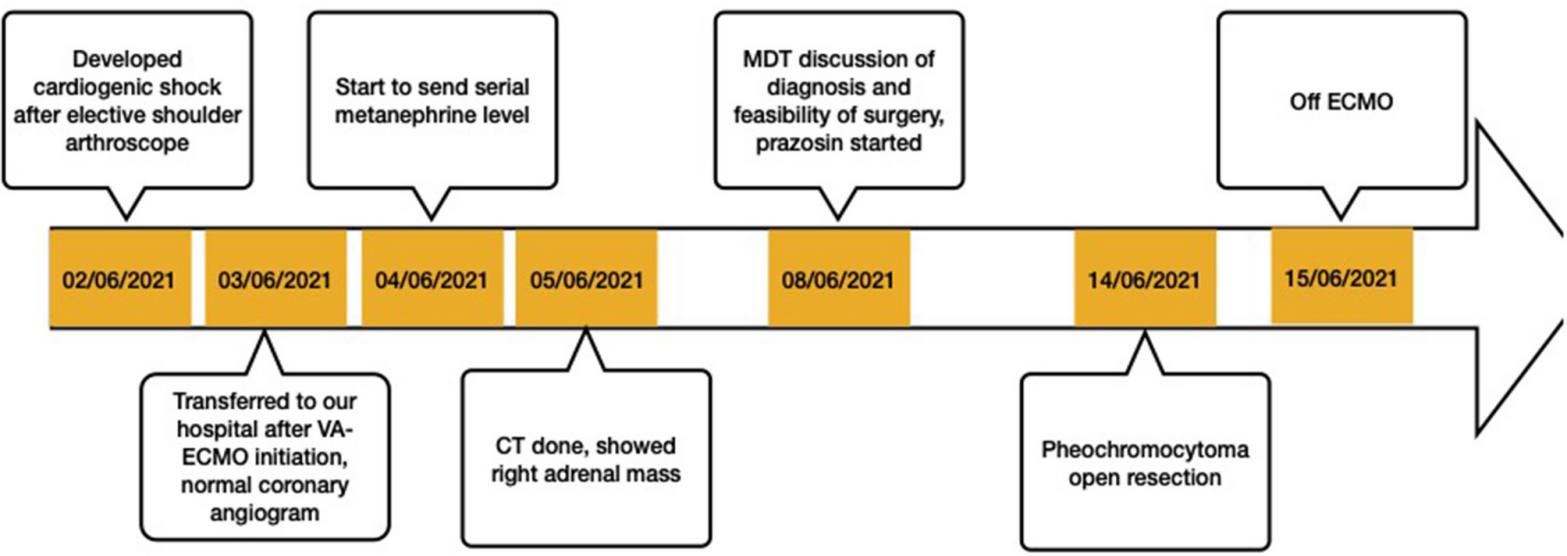
The timeline of case progression.

Subsequently, a multidisciplinary team (MDT) discussion, including an endocrinologist, a cardiologist, a urologist, and an intensivist, was held. The patient's clinical history and investigations were reviewed, and diagnosis of pheochromocytoma-induced cardiomyopathy was made during the MDT discussion. There was also detailed discussion about the timing of pheochromocytoma resection. The consensus was to start an alpha adrenergic blocker gradually and aim for an early surgery. Prazosin was started at a dosage of 0.5 mg two times a day, and increased daily by 1 mg. After 6 days of alpha blockade preparation, with prazosin of 3 mg two times a day, the patient underwent an uneventful open right pheochromocytoma resection with ongoing VA-ECMO support. Heparin was ceased 6 h prior to surgery with activated clotting time at 110 s prior to the surgery and was resumed 12 h post-operatively. ECMO was removed 1 day later, and the patient was extubated successfully on post-operative Day 5. Figure 3B is the histological image of the adrenal tumour. The sample was well-circumscribed and unencapsulated, was a size of 2 cm × 1.5 cm × 1 cm, and it showed a vaguely nested architecture and vesicular overlapping nuclei. The immunohistochemical examination of the specimen showed appearance typical of pheochromocytoma, with specific neuroendocrine markers, including chromogranin A, a neural cell adhesion molecule, and synaotophysin, all returned positive. She was transferred to a rehabilitation centre after 24 days of stay in the intensive care unit (ICU). Upon transfer, her LVEF recovered to 55% as compared to 20% on admission. Figure 4 is a timeline of the clinical condition progress and major management of the patient.

## Discussion

According to the guideline, pheochromocytoma should be excluded before making the diagnosis of stress-induced cardiomyopathy (3). A large registry study demonstrated that more than 90% of patients with stress-induced cardiomyopathy were female at the age of 50s or older with neurologic disorder and emotional stress as common triggers (4). After a normal coronary angiogram, our patient seemed to well-fit in the diagnosis of stress-induced cardiomyopathy, given her age and possible stress from the surgery. However, the exclusion criteria of stress-induced cardiomyopathy reminded us to rule out pheochromocytoma before making a diagnosis of stress-induced cardiomyopathy. Indeed, further history of adrenal mass raised up the suspicion for pheochromocytoma-induced cardiomyopathy, which later proved to be the case. In the review paper by Agarwal et al. (1), they compared patients with pheochromocytoma-induced cardiomyopathy and stress-induced cardiomyopathy. The results showed that the patients with pheochromocytoma-induced cardiomyopathy were at higher risk of developing cardiogenic shock. They proposed that, in patients diagnosed with stress-induced cardiomyopathy, even in the absence of signs and symptoms of pheochromocytoma, a workup of pheochromocytoma should be strongly considered. This case again reminds us that every effort should be made to exclude pheochromocytoma in patients who present with features of stress-induced cardiomyopathy, especially those who present with cardiogenic shock.

A new diagnosis of pheochromocytoma in critically ill patients can be challenging. The usual diagnosis of pheochromocytoma includes biochemical testing as well as imaging (3). A previous study has shown that the plasma-free metanephrine level is a reliable test for confirming pheochromocytoma (5). In the critical care setting, the interpretation of catecholamines is more challenging due to the use of adrenergic agents as well as endogenous (physiological) release. A retrospective study comparing urine metanephrine levels from inpatient patients (including 132 patients from ICU) without pheochromocytoma with those with confirmed pheochromocytoma showed that there was a significant overlap of urine normetanephrine and metanephrine levels between hospitalised patients and patients with pheochromocytoma (6). The study concluded that for, ICU patients, cathecholamine levels more than five-fold above the upper reference limit had 98% specificity for the diagnosis of pheochromocytoma, but still had poor positive predictive value. As our patient was still on noradrenaline, the normetanephrine level was possibly affected by exogenous infusion, but no adrenaline was used since admission to our hospital, so the metanephrine level was unlikely affected by exogenous factors. As the patient was anuric, urine measurement of normetanephrine and metanephrine was deemed impossible; hence, the biochemical diagnosis, in this case, was based on the serum metanephrine level. The result showed that the baseline level of metanephrine level for this patient was already at least five times over the upper limit of the reference range. And, during the two episodes of the sudden onset of hypertension and tachycardia, the metanephrine level was near 20 times over the upper limit. This was the biochemical basis of diagnosis for pheochromocytoma in this patient. Together with the typical contrast CT findings, the diagnosis of pheochromocytoma-induced cardiomyopathy was made during the MDT discussion.

In our case, the patient was initiated on VA-ECMO support for isolated left ventricular failure with preserved right ventricular function. As we have known, VA-ECMO support can provide circulatory support, but it is unlikely to improve left ventricular function (7). In fact, it can cause detrimental effects to the left ventricle as they increase left ventricle after load and stress. From this perspective, axial left ventricle-assisted devices, such as IMPELLA or TandemHeart would be more favourable. However, from practical aspects, axial left ventricle assisted-devices are not available in our institution unfortunately. For our case, the patient had cardiac arrest before VA-ECMO initiation; the preference for a mechanical circulatory support device would be one can provide biventricular support as there was still high risk of recurrence of cardiac arrest, especially with an unclear primary pathology causing cardiogenic shock. An axial left ventricle device will not be sufficient if the patient went into cardiac arrest again.

Another challenge of managing this case was the timing of surgery. Guidelines recommend that all patients with pheochromocytoma should receive adequate alpha- and beta-blockade pre-operatively to prevent perioperative cardiovascular complications (3). While this fits well for elective resection of pheochromocytoma in stable patients, it was difficult to apply to our patient who suffered refractory cardiogenic shock and cardiac arrest, requiring mechanical circulatory support due to pheochromocytoma. Whilst all specialists including the anaesthetist agreed that the surgery should be done early, there were a few concerns of performing surgery for this patient. The first was whether to have the surgery done whilst the patient was still on VA-ECMO support. The benefit of performing pheochromocytoma resection surgery whilst on VA-ECMO support was ECMO can provide cardiac support in case the patient developed worsening cardiogenic shock or cardiac arrest during surgery due to a possible catecholamine surge during surgery. However, anticoagulation had to be ceased peri-operatively, which can increase the risk of ECMO-related complications, such as thrombotic events and oxygenator failure. Another concern was, without gradual and adequate adrenoreceptor blockade, the patient might develop hypertensive crisis from a catecholamine surge, which could result in complications, such as cerebrovascular accidents. Also, without letting the body gradually adapt to the “decreased” catecholamine levels by adrenoreceptor blockade, the patient might develop severe vasoplegic shock due to sudden decreased catecholamine levels post-operatively, compounded by pharmacological adreno-receptor blockade. In this patient, we successfully introduced a short-acting alpha blocker, whilst the patient was still on VA-ECMO support. The patient subsequently underwent surgery uneventfully 6 days later, whilst most case series and case reports proposed that it might be safer to introduce alpha and beta blockers and perform adrenalectomy several weeks after the initial catastrophic presentation for pheochromocytoma-induced cardiogenic shock managed on VA-ECMO (8, 9). We managed to safely introduce alpha blockers in pheochromocytoma-induced cardiomyopathy whilst patient was in cardiogenic shock requiring ECMO and subsequently performed early pheochromocytoma resection with VA-ECMO support.

There are a few limitations of our case report. First, this is a single case report of pheochromocytoma resection under VA-ECMO support after an accelerated preparation. Second, we were unable to perform MIBG (Metaiodobenzylguanidine) scintigraphy pre-operatively to further confirm the diagnosis and rule out possibility of metastatic pheochromocytoma/paraganglioma, whilst the patient is on VA-ECMO support. And we did not follow up the patient further after she was transferred to the rehabilitation facility. The future direction would be further exploration of optimal duration of alpha and beta-adrenergic blockade therapy if patient is deemed requiring for an accelerated pre-operative preparation.

In conclusion, we presented a unique case with refractory cardiogenic shock due to pheochromocytoma-induced cardiomyopathy without a known history. Although rare, this case highlighted the importance of excluding patients with pheochromocytoma with features of stress-induced cardiomyopathy, especially those with cardiogenic shock. And early removal of pheochromocytoma after an accelerated preparation pre-operatively was feasible with VA-ECMO support.

## Data Availability Statement

The raw data supporting the conclusions of this article will be made available by the authors, without undue reservation.

## Ethics Statement

Written informed consent was obtained from the relevant individual's legal guardian/next of kin, for the publication of any potentially identifiable images or data included in this article.

## Author Contributions

TiL and JN collected the data and participated in manuscript writing. ZL and ToL participated in manuscript writing. All authors read and approved the final manuscript.

## Conflict of Interest

The authors declare that the research was conducted in the absence of any commercial or financial relationships that could be construed as a potential conflict of interest.

## Publisher's Note

All claims expressed in this article are solely those of the authors and do not necessarily represent those of their affiliated organizations, or those of the publisher, the editors and the reviewers. Any product that may be evaluated in this article, or claim that may be made by its manufacturer, is not guaranteed or endorsed by the publisher.

## Abbreviations

VA-ECMO: veno-arterial extracorporeal membrane oxygenation
ECG: electrocardiogram
STEMI: ST-segment-elevated myocardial infarction
LVEF: left ventricular ejection fraction
ROSC: return of spontaneous circulation
MDT: multidisciplinary team.
